# Respiratory and muscular effort during pre-slaughter stress affect Nile tilapia fillet quality

**DOI:** 10.1371/journal.pone.0306880

**Published:** 2024-07-12

**Authors:** Silvia Prestes dos Santos, Maria Ildilene da Silva, Antonio Cesar Godoy, David Geovanni De Almeida Banhara, Marcio Douglas Goes, Elenice Souza dos Reis Goes, Claucia A. Honorato

**Affiliations:** Faculdade de Ciências Agrárias, Universidade Federal da Grande Dourados, Dourados, MS, Brazil; ICAR Indian Institute of Agricultural Biotechnology, INDIA

## Abstract

Nile Tilapia (*Oreochromis niloticus*) management procedures are directly linked to the final quality of the product. The aim of this study was to evaluate the effect of pre-slaughter density and different stunning methods on biochemical, respiratory and muscle injury parameters associated with quality and sensory characteristics of Nile tilapia fillets. Fish with an average weight of 762±105 g were used, first collected called the control group. The experiment was conducted in a 2 × 2 factorial scheme, with two densities (50 and 300 kg of live weight m^−3^) and two stunning methods thus totaling four treatments, with 15 repetitions per treatment totaling 75 fish sampled. Blood gas analysis, evaluation of biochemical parameters, analysis of meat quality and sensory analysis were carried out. For blood gas, biochemical and enzymatic parameters, the highest values were obtained for the density of 300 kg m^−3^ and asphyxia method: partial pressures of CO2; glucose and lactate, the highest values presented were 268.98 and 11.33 mg dL^−1^ respectively. As well as enzymatic activities, Creatinine kinase (CPK); Creatinine kinase isoenzyme (CKMB) showed higher values (768.93 and 1078.98 mg dL^−1^ respectively) in the higher density and asphyxia method. Conversely, when evaluating the quality parameters, the highest values were observed for lower density and thermonarcosis. High depuration density (300 kg m^−3^), combined with the asphyxiation stunning method, promotes changes in respiratory dynamics and provides greater stress, less firm fillet texture and greater weight loss due to cooking, as well as changes in creatine kinase (CK) and its CK-MB isoenzyme, demonstrating greater muscle damage. On the other hand, the density of 50 kg m^−3^ during pre-slaughter, combined with the method of stunning by thermonarcosis, provide a longer period of permanence in pre rigor mortis, which will result in fillets with a better sensory profile.

## Introduction

Tilapia (*Oreochromis niloticus*) is a globally prevalent species in aquaculture [1], known for its high production rates [2], primarily utilized in fillet production [3]. Contributing to 75% of global aquaculture production, Nile tilapia is an affordable and healthy protein source [4, 5]. It is the third most important fish species raised in captivity worldwide, with a total production of approximately 5.3 million tonnes [6]. The intensive aquaculture of Nile tilapia is rapidly expanding due to the increasing global demand.

Some studies report the effect of stress on fish products quality [7, 8] and these studies have been looking for molecular biochemical markers [9, 10] capable of previously identifying the quality of the fish that will be slaughtered.

Pre-slaughter transport can lead to stress, resulting from increased physical activity [11, 12]. Thus, it is essential to carry out the resting process to restore these functions [13]. Also, the way of stunning the fish, such as thermonarcosis [14] and asphyxiation [15], and the method of slaughter [16], can promote a decrease in energy reserves [12]. Also, the change of environment causes high stress in the animals, causing an increase in swimming activity and this condition in the period before slaughter alters the respiratory responses [8], promoting an increase in plasma cortisol, glucose and chloride [17] and anaerobiosis, with a substantial increase in lactic acid and a faster decrease in muscle pH, thus altering the onset of rigor mortis [18]. Such biochemical alterations, causing losses in the quality of the final product, such as ruptures in connective and muscular tissues [19, 20], changes in color [21] juiciness [22], tenderness [10] and reduction [23, 24] water holding capacity, reducing the shelf life [23, 25].

It is evident that animal welfare is gaining increasing importance in animal production systems. Welfare assessment protocols now encompass criteria such as nutrition, health, environment, and the expression of natural behaviors to ensure overall well-being [26]. Careful handling of fish during removal from breeding areas, transport, and the period prior to slaughter significantly impacts their well-being, which, in turn, can influence the sensory quality of the fillet [9, 13].

The stocking density of fish in the resting period [7], resting time [13] and the stunning method [9] can be a source of stress, promoting physiological changes that result in changes in blood metabolism [27], causing insufficient gas exchange, changes in electrolyte balance and changes in plasma stress markers [8].

Current research has concentrated on the impact of live-holding transport on the antioxidant status of fish. However, there is a lack of studies exploring the correlation between different slaughter methods and the antioxidant status of fish fillets, as well as protein oxidation [28]. Inadequate slaughtering procedures can significantly increase stress levels in fish, accelerating the onset of rigor mortis. Pre-slaughter stress leads to elevated lactic acid levels, which lower the pH in fillets. This rapid pH decline causes myosin protein denaturation and alters solubility, resulting in reduced water-holding capacity (WHC). Consequently, it promotes protein degradation, lipid oxidation, and microbial growth [29]. There is a relationship between slaughter methods and the cardiac status of fish fillets and sensory effects and recent studies on fish pre-slaughter protocols have increasingly focused on minimizing stress to improve fillet quality [30]. Techniques such as reducing handling time and using less invasive stunning methods have been demonstrated to reduce muscle rigidity and improve fillet texture and flavor, thereby contributing to a higher quality end product [31].

Studies with the aim of evaluating the effect of stress arising from pre-slaughter handling on the physiological parameters of the animals can bring benefits to the aquaculture industry, as it will enable fish in *natura* and derivatives with higher quality and that it will adopt more appropriate techniques prioritizing homeostasis, making the fish production process more humanized. Therefore, this work aimed to evaluate the effect of pre-slaughter density and different stunning methods (thermonarcosis and asphyxia) on hemogasometric analysis, Blood biochemical parameters evaluation, meat quality analysis and sensory characteristics of Nile tilapia filets.

## Materials and methods

This trial was carried out in accordance with the guidelines of the Brazilian College of Animal Experimentation (COBEA; http://www.cobea.org.br) and was approved by the Animal Care Committee of the Universidade Federal da Grande Dourados (CEUA), under protocol No. 43/2016 –UFGD/Brazil.

### Animals, transport and experimental groups

Nile tilapia (*O. niloticus*) with an average weight of 762±105g were used. As an initial sample to verify homeostasis, 15 fish were collected under the same minimum stress conditions as the other fish sampled, totaling five treatments. Initially, the animals were removed from the excavated ponds with the aid of a net and placed in a transport box with constant aeration, at a stocking density of 200 kg m^−3^.

The experiment was conducted in a factorial scheme 2×2, with two densities (50 and 300 kg of live weight m^−3^) and two stunning methods (thermonarcosis and asphyxia) with 15 repetitions per factor (each fish being an experimental unit) in a total of 60 fish sampled. 6.0 g L^−1^ sodium chloride (NaCl) was added to the transport water, and the water temperature was lowered using ice to 21°C. The fish were transported for one hour, until they arrived at the Agricultural Products Analysis Laboratory of the Faculty of Agricultural Sciences of Federal University of Grande Dourados.

After the fish arrived at the laboratory, the animals were placed in polyethylene boxes with a capacity of 500 L, with an artificial aeration system, using two densities (50 and 300 kg of live weight m^−3^). The fish remained at these densities for one hour of rest and, after that, 30 fish were randomly sampled per density, and 15 fish were destined for stunning by thermonarcosis in water and ice (1:1–0.0°C) and 15 fish were destined to the asphyxiation stunning method. The control group was euthanized on the property by sectioning the marrow.

### Parameters analyzed

#### Hemogasometric analysis

For hemogasometric analysis, 10 fish per treatment were used. The blood was analyzed by automation in a Cobas HB121 hemogasometer ‐ Roche Diagnostica Brasil, São Paulo, SP, Brazil. The ions contents were measured (nmol L^−1^): sodium (Na^+^), potassium (K^+^) and chloride (Cl^−^). Respiratory parameters were also determined in fish blood pH (1–10), H_3_O^+^, bicarbonate concentration (HCO_3_^−^), oxygen partial pressure (PO_2_) and carbon dioxide partial pressure (PCO_2_) and functional oxygen saturation (SO_2_) and total volatile bases according to Ashwood et al. 1983 [32] and Mandelman et al. 2009 [33].

#### Blood biochemical parameters evaluation

For the biochemical analyzes of glucose, protein, creatinine, albumin, lactate, Creatinine kinase (CK) and its CK-MB isoenzyme, whole blood samples were obtained from 10 specimens per treatment with the aid of 3.0 mL syringes and needles (Gauge) previously bathed in heparin.

Glucose concentration was determined using an Accu-Chek Advantage II–Roche electronic meter, using 10 *μ*L blood samples. Total protein, creatinine and albumin were estimated using the Gold Analisa Diagnóstica kit, with reading by spectrophotometry (BIO PLUS S 200). The kit for determination of creatinine uses the colorimetric method Alkaline Picrate–Jaffé.

The lactate concentration was measured by Lactate Kit (Katal Biotecnológica Ind. Com. Ltda. Minas Gerais, MG, Brazil) and CK and CK-MB the kinetic UV method [34]. Protein concentrations in crude enzyme extracts were determined as [35], at 450 nm, and 1.0 mg mL^−1^ of albumin as standard.

The activity of liver CAT and SOD, fragments of the liver were homogenized in a buffer solution with 10 mM potassium phosphate, pH 7.0 (1:10), and centrifuged at 15,000 rpm at 4°C during 20 min. After this period, the supernatant was transferred to microcentrifuge tubes and maintained at -70°C. CAT activity was evaluated in a spectrophotometer following the method described by [36] which consists of measuring CAT activity by measuring the consumption of exogenous hydrogen peroxide (H_2_O_2_) with generation of oxygen and water. For SOD it was adapted to the methodology of Mccords and Fridovich, 1969 [37].

#### Meat quality analysis

Meat quality analyzes were evaluated in 10 specimens per treatment considering pre- *rigor mortis* time (PRMT), pH, water holding capacity (WHC), cooking water loss (CWL) and tenderness. It was evaluated in terms of time (min) from slaughter to entry into *rigor mortis*. The evaluations were carried out every 20 minutes, until the fish reached the accuracy index of 100%. The rigor index (RI) was measured according to Bito [38], and calculated according to the Equation 1: RI = ((D_0_-D]/D_0_)*100 (Where: D_0_ = value of the distance separating the base of the caudal fin from the reference point, immediately after death and D = value of the distance separating the base of the caudal fin from the reference point in the selected time intervals).

The pH was measured in triplicate per fillet, in ten fish per treatment, 24 hours *post-mortem*, using a portable digital pH meter (Testo^®^ model 205), with an electrode inserted for meat. As for the water holding capacity (WHC) parameter, it was performed according to Barbut [39], in triplicate, in ten fish from each treatment, 24 hours *post-mortem*. For this purpose, 0.5 g meat samples were placed between two circular qualitative filter papers measuring 5.5 cm in diameter, 205 *μ*m thick and weighing 80 g m^−2^, placed between two square glass plates with a thickness of 8 mm each. Uniform pressure was applied to this set using a 10 kg weight for five minutes. Afterwards, the samples were weighed again and the difference between final and initial weight was expressed as a percentage.

The measurement of water loss due to cooking was carried out 24 hours *post-mortem*, according to Cason et al. [40]. Exactly 70.0 g of meat were weighed, placed in plastic bags and cooked in a bain-marie until the internal temperature reached 75.0 to 80.0°C, through monitoring with a digital thermometer. Afterwards, the samples were cooled down to 30.0°C, and weighed again. The difference between the initial and final weight was expressed as a percentage, corresponding to the water loss due to cooking.

Color measurements were performed 24 hours *post-mortem*, on the ventral face of the fillet, taking six different reading points per sample, in ten fish per treatment. Brightness (L*) values were evaluated using a colorimeter (Minolta^®^ CR-400) at a 90° angle. The L* values represent the brightness scale, ranging from 0 (black) to 100 (white). In addition, chroma a* (red-green component) and chroma b* (yellow-blue components) were measured to provide a comprehensive color profile. All measurements were performed at room temperature. The tenderness analysis of the fillets was carried out 24 hours post-mortem, by measuring the shear force. Prior to analysis, raw meat samples were left at room temperature for approximately one hour. For this evaluation, a Stable Micro Systems Texture Analyzer texturometer (model TA-XT Plus) was used, equipped with an SMS shear cell (Stable Micro Systems), Guillotine Blade (USDA), with a thickness of 3.00 mm, length 70.0 mm and angle from 90°. The fillet samples were cut into cubes transversely from the direction of the muscle fibers in measures of approximately 20.0 × 25.0 × 20.0 mm. The analysis was performed in triplicates per fillet of ten fish per treatment, obtaining the shear force parameter in Newton per cm^2^ (N cm^−2^).

#### Sensory analysis

72 hours after the fish were slaughtered, samples (kept under refrigeration ≈5.0°C) of fillets cut into cubes (±3.0 g), packed in aluminum foil and subsequently submitted to an electric grill, until internal meat temperature reaching 76.0°C were offered to 100 untrained tasters, following the [41] protocol. Women represented 54% of the panelists and men 46%. The predominant age group was 21 to 30 years old (62%), followed by under 20 years old (31%), 41 to 50 years old (5%) and finally 51 to 60 years old (2%). Volunteer tasters were asked to rate each sample on the acceptability of four attributes (color, texture, juiciness, and overall acceptability) using a 9-point scale, ranging from 1 (dislike extremely) to 9 (like extremely). Consumers were asked to eat crackers and rinse their mouths with water before evaluating each sample, including the first sample.

#### Statistical analyses

Statistical analyses were performed using R software [42]. Homogeneity of variance of production performance and liver parameter data was tested using the Shapiro-Wilk test [43]. The Levene test [44] was used to test for normality. The data were then subjected to two-way ANOVA followed by Tukey’s post hoc test. The results were expressed as mean and standard deviation. For hedonic scale data, the non-parametric Kruskal-Wallis test through the Agricolae package [45] using R software [42] was used to identify differences between treatments. Significant differences were considered when p < 0.05.

## Results

The association between the depuration density (50 and 300 kg m^−3^) and the asphyxia stunning method promoted changes in respiratory dynamics in relation to the control group (Table 1).

**Table 1:** Hemogasometric parameters of *O. niloticus* submitted to different stocking densities (50 and 300 kg m^−3^) and stunning methods (thermonarcosis and asphyxia).

Stocking density (kg/m³)	Stunming method	pH	^1^PCO_2_ (mmHg)	^2^PO_2_ (mmHg)	^3^SO_2_ (%)	^4^C_HCO3_^-^ (mmol L^−1^)	^5^BE
50	Asphyxy	7.20 ±0.11	36.32 ±1.11*	93.49 ±2.20	80.11 ±2.23	12.77 ±1.93	-22.45 ±0.83*
	Thermonarcosis	7.07 ±0.07	28.00 ±1.93	107.85 ±39.71	65.88 ±7.40	12.03 ±1.65	-15.15 ±1.75
300	Asphyxy	6.77 ±0.09*	45.50 ±5.58*	87.65 ±20.45	75.70 ±6.37	7.80 ±0.65	-24.33 ±0.74*
	Thermonarcosis	7.09 ±0.16	36.90 ±3.43*	58.94 ±23.52	64.08 ±9.22	14.62 ±3.08	-18.35 ±1.91
Control		7.26 ±0.03	25.34 ±1.37	98.56 ±32.03	60.90 ±7.41	11.17 ±0.82	-15.36 ±0.70
Stocking density (kg/m³)							
50		7.14 ±0.07	32.16 ±1.64b	100.67 ±19.08	73.00 ±4.27	12.40 ±1.22	-18.80 ±1.44
300		6.93 ±0.10	41.20 ±3.38a	73.29 ±15.48	69.89 ±5.62	11.21 ±1.82	-21.34 ±1.33
Stunming method							
Asphyxy		6.98 ±0.09	40.91 ±3.05a	90.57 ±9.84	77.90 ±3.29	10.28 ±1.23	-23.39 ±0.60b
Thermonarcosis		7.08 ±0.08	32.45 ±2.31b	83.39 ±23.21	64.98 ±5.64	13.33 ±1.71	-16.75 ±1.32a
Dunnet test		p<0.05	p<0.05	p>0.05	p>0.05	p>0.05	p<0.05
p-value							
Stocking density x stunning effect		0.0635	0.9677	0.4041	0.8500	0.0768	0.6450
Stocking density effect		0.0787	0.0166	0.2914	0.6533	0.5624	0.0860
Stunning effect		0.4046	0.0239	0.7792	0.0722	0.1483	0.0001

* Means differ from the control treatment by Dunnet’s test (p < 0.05). Means in the same column followed by different letters differ with each other by the Tukey test (p < 0.05). 1 ‐ Partial pressure of carbon dioxide; 2 ‐ Partial Pressure of Oxygen; 3 ‐ Oxygen saturation; 4 ‐ Bicarbonate concentration; 5 ‐ Volatile bases.

At a depuration density of 300 kg m^−3^, the fish remained in hypoventilation due to the high values of PCO_2_, and the stunning method by asphyxiation presented the highest values, as well as demonstrating compensatory respiratory acidosis, since the pH maintained the homeostatic control with its buffer function, together with the stability of the bicarbonate (CHCO_3_^−^) and also in association with the decrease of bases.

When evaluating the stocking density of 300 kg m^−3^ and the asphyxiation stunning method, it is verified that the association between these two parameters promotes the increase of glycemia and blood lactate, as the highest values for asphyxia at the density of 300 animals m^−3^. All means were higher (p< 0.05) than the control. Increased creatinine and CPK and CKMB activity were observed in the stocking density of 300 kg m^−3^ and the use of the asphyxiation stunning method. The meat quality parameters demonstrated that the interaction between 300 kg m^−3^ density and the asphyxia stunning method promoted fillets with greater Cooking Weight Loss (CWL).

In relation to the other treatments. The density of 50 kg m^−3^ provided fillets with greater intensity of red, greater shear force, longer pre-rigor time, and lower PPC compared to fillets of fish submitted to the density of 300 kg m^−3^. The stunning by asphyxia led to the development of fillets with greater brightness, lower yellowness, lower shear force, lower water holding capacity (WHC), and shorter pre-rigor mortis time when compared to fish stunned by thermonarcosis (Table 2).

**Table 2:** Biochemical parameters of *O. niloticus* submitted to purification at different stocking densities (50 and 300 kg m^-3^) and stunning methods (thermonarcosis and asphyxia).

Stocking density	Stunning method	Glucose	Lactate	Protease	Albumin	Creatinine	^1^CPK	^2^CKMB
50	Asphyxy	190.80 ±12.98b*	4.78 ±0.92b*	11.14 ±0.30*	1.42 ±0.11	0.51 ±0.02*	519.95 ±27.97*	809.52 ±36.89*
	Thermonarcosis	178.25 ±14.39b*	1.97 ±0.26bc	13.29 ±0.72*	1.87 ±0.07	0.74 ±0.08*	142.73 ±21.22*	478.93 ±12.39
300	Asphyxy	268.98 ±24.91a*	11.33 ±1.11a*	12.35 ±1.45*	1.72 ±0.26	0.55 ±0.11*	768.93 ±32.02*	1078.98 ±46.06*
	Thermonarcosis	133.77 ±12.72b*	1.48 ±0.38c	13.31 ±1.01*	2.02 ±0.16	0.61 ±0.08*	292.33 ±43.90	735.77 ±64.27*
Control		91.16 ±8.12	1.13 ±0.11	4.17 ±0.21	1.56 ±0.06	0.22 ±0.03	262.40 ±16.55	549.12 ±35.30
Stocking density (kg m^-3^)								
50		184.53 ±9.43	3.37 ±0.62b	12.21 ±0.49	1.64 ±0.09	0.62 ±0.05	331.34 ±59.28b	644.23 ±53.18b
300		201.38 ±24.36	6.40 ±1.59a	12.83 ±0.85	1.87 ±0.15	0.58 ±0.07	530.63 ±76.38a	907.38 ±64.02ª
Stunning method								
Asphyxy		229.89 ±17.84a	8.05 ±1.20a	11.74 ±0.73	1.57 ±0.14b	0.53 ±0.05	644.44 ±42.66a	944.25 ±49.41ª
Thermonarcosis		156.01 ±11.35b	1.72 ±0.23b	13.30 ±0.59	1.94 ±0.08ª	0.67 ±0.06	217.53 ±32.39b	607.35 ±49.73b
Teste de Dunnet		p<0.05	p<0.05	p<0.05	p>0.05	p<0.05	p<0.05	p<0.05
p-value								
Stocking density x stunning effect		0.0018	0.0002	0.5421	0.6645	0.3259	0.1401	0.8874
Stocking density effect		0.3338	0.0007	0.5311	0.1885	0.5743	0.0001	0.0001
Stunning effect		0.0003	0.0001	0.1223	0.0350	0.0842	0.0001	0.0001

* Means differ from the control treatment by Dunnet’s test (p < 0.05). Means in the same column followed by different letters differ with each other by the Tukey test (p < 0.05). 1 ‐ Creatinine kinase; 2 ‐ Creatinine kinase isoenzyme.

The averages of the factorial scheme obtained for pH, brightness, and WHC in fillets in the control group did not differ (p> 0.05) from those in other groups. The sensory attributes analysis showed that the interaction between the stocking density and the stunning method was not significant (p> 0.05) (Table 3). However, evaluating the stunning method separately, different values were observed for the attributes of juiciness (p< 0.01) and general acceptability (p> 0.05); the stunning by thermonarcosis provided fillets with higher scores in these attributes.

**Table 3:** Quality parameters of *O. niloticus* fillets submitted to depuration at different stocking densities (50 and 300 kg m^−3^) and stunning methods (thermonarcosis and asphyxia).

Stocking density	Stunning method	PRM (min)	pH	Brightness	Chroma a	Chroma b	Shear force (×10 N cm^−2^)	WHC (%)	CWL (%)
50	Asphyxy	190 ±20*	6.87 ±0.04	39.79 ±0.40	-0.02 ±0.10*	-2.61 ±0.21	3.38 ±0.32	54.17 ±0.35	4.48 ±0.20b*
	Thermonarcosis	325 ±25	6.68 ±0.04	36.92 ±0.63	0.25 ±0.18*	-1.70 ±0.29*	3.93 ±0.22	56.02 ±0.67	5.65 ±0.39b
300	Asphyxy	54 ±11*	6.86 ±0.05	39.86 ±0.41	-0.27 ±0.10	-2.54 ±0.23	2.64 ±0.32*	54.38 ±0.51	7.19 ±0.45ª
	Thermonarcosis	289 ±56	6.86 ±0.06	37.83 ±0.39	-0.18 ±0.11	-2.41 ±0.17	3.52 ±0.29	56.43 ±0.51	5.52 ±0.24b
Control		438 ±55	6.77 ±0.04	38.39 ±0.57	-0.52 ±0.10	-2.66 ±0.15	4.32 ±0.20	56.13 ±0.72	6.79 ±0.39
Stocking density (kg m^-^³)									
50		250 ±28ª	6.77 ±0.04	38.35 ±0.49	0.11 ±0.11a	-2.15 ±0.20	3.66 ±0.20a	55.10 ±0.47	5.01 ±0.27
300		184 ±51b	6.86 ±0.04	38.90 ±0.37	-0.23 ±0.07b	-2.48 ±0.14	3.06 ±0.23b	55.41 ±0.48	6.35 ±0.37
Stunning method									
Asphyxy		129 ±27b*	6.86 ±0.03	39.83 ±0.28a	-0.15 ±0.07	-2.58 ±0.15a	3.01 ±0.24b	54.28 ±0.29b*	5.71 ±0.48
Thermonarcosis		305 ±32ª	6.76 ±0.04	37.35 ±0.38b	0.05 ±0.12	-2.03 ±0.19b	3.73 ±0.18a	56.23 ±0.40ª	5.58 ±0.22
p-value									
Stocking density x stunning effect		0.1812	0.052	0.381	0.490	0.104	0.5790	0.8537	0.0004
Stocking density effect		0.0298	0.077	0.303	0.013	0.175	0.0434	0.5597	0.0009
Stunning effect		0.0001	0.054	0.000	0.160	0.031	0.0193	0.0018	0.4518

Brightness (0 black and L = 100 white), chroma a (red-green component) and chroma b (yellow-blue component). WHC: Water holding capacity. CWL: Cooking Weight Loss. Means in the same column followed by different letters differ by Tukey’s test.

*Means differ from the control treatment by Dunnet’s test (p< 0.05); Data expressed as mean ± standard error.

**Fig 1:**
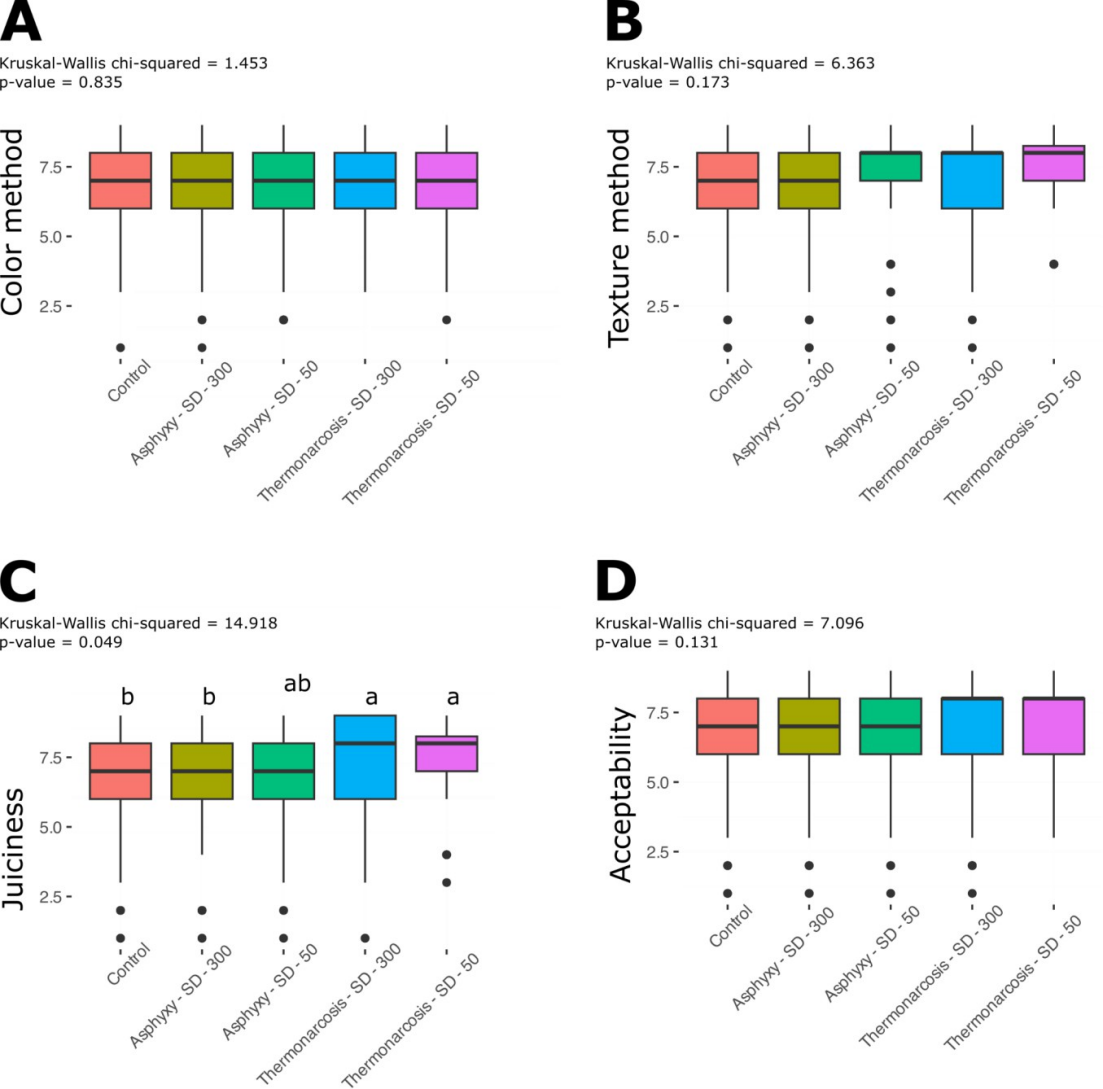
Sensory profile of *O. niloticus* fillets submitted to different densities (50 and 300 kg m^−3^) and different stunning methods (thermonarcosis and asphyxia). Hedonic scale between 1 (dislike extremely) and 9 (like extremely). Kruskal-Wallis H test. Different letters indicate statistically significant (p < 0.05) differences between treatment groups.

The medians of the sensory attributes at the two storage densities (50 and 300 kg m^-3^) and stunning methods (thermonarcosis and asphyxia), compared with the means of the control treatment, showed that the quality of color, texture and general acceptability were equivalent (p>0.05) to the medians of the control treatment (Fig 1A, 1B and 1D). On the other hand, the median juiciness presented significantly higher values for the asphyxiation treatments at a density of 50 kg m^-3^ and thermonarcosis at densities of 50 and 300 kg m^-3^ (p<0.05) (Fig 1C).

## Discussion

The stocking density during rest can influence the well-being of the fish, restoring the physiological characteristics before transport management. Management studies are of paramount importance to optimize the fish production chain [7, 46, 47]. In this essay, we demonstrate that stocking density can influence respiratory function and have consequences on homeostasis. The present study provided novel findings regarding the elevated levels of enzymes in the heart muscle, indicating significant muscle damage as a result of the high stocking density and the stunning method used. However, the association with the stunning method that the fish are subjected to can exacerbate the adverse effects having consequences on meat quality [7, 15].

The increase in ventilatory responses was not enough to maintain oxygen dynamics right after transport and also in fish submitted to a stocking density of 300 kg m^−3^, resulting in acidosis [47], despite the increase in HCO_3_^−^ in the blood. The fish, after transport and at a stocking density of 50 kg m^−3^, remained at baseline values, suggesting that the acid-base balance was restored. It is noteworthy that the stocking density is effective for reestablishing homeostasis, demonstrated by the blood pH values close to neutrality. The acid-base balance is important [7], having a direct relationship with respiratory functions [48] and consequently energy expenditure [47].

One of the most pronounced changes in transport-associated management is plasma glucose [46]. Stress-related hyperglycemia is described for several species of fish [7, 9, 13] and is a common alteration to transport [46, 48]. The restoration of glucose levels to baseline values is required to guarantee the quality of the fish to be marketed [13, 49]. In the stunning method by asphyxiation, the fish showed hyperglycemia in relation to the stunning by thermonarcosis due to the increase in glucocorticoids, corticosteroids and catecholamines that raise the blood sugar level [50]. Glycogen consumption was increased [51, 52] in asphyxia because it promotes slower death [53, 54], due to the longer movement time in anaerobic conditions.

Thermonarcosis is widely used for several species of fish, especially for tropical fish, as rapid cooling results in a rapid interruption of fish movement [54]. However, several studies point out that, although physical reactions stop or slow down quickly in ice, brain activity indicates the continuation of consciousness for a substantial period [11, 55, 56]. Changes in creatinine were observed in situations that provided greater movement and increased plasma glucose. Creatinine is a muscle tissue metabolite, indicative of muscle protein catabolism [57]. Fish subjected to stressful conditions tend to mobilize energy sources by raising muscle lactate concentrations [58] and serum creatinine [46].

The increase in creatinine, as an indication of muscle protein catabolism, may indicate that fish in conditions of high stocking density require greater swimming effort, and in situations of hypoxic stress, it promotes more severe damage to the muscles [59]. These results are supported by the greater activity of CK and CKMB observed in fish in this treatment. The CK and CKMB activities also increased in *Hypophthalmichthys molitrix* under hypoxia [59] and in animals under fatigue [60]. Elevated serum creatine kinase levels are indicative of stress, muscle damage and muscle fatigue [61]. Stunning methods involving increased physical activity before death, combined with pre-slaughter stress, lead to the consumption of the glycogen energy reserve at the expense of adenosine triphosphate (ATP), at the same time that lactic acid production occurs in the muscle [62] promoting muscle injuries verified by the presence of CK and CKMB [60]. By the asphyxiation method, the fish takes a long time until it is dead, which provokes vigorous movements and agitation behavioral responses in the animals [21], resulting in a greater expenditure of energy reserve (ATP) before death and a shortened period permanence in pre-*rigor mortis*.

This condition was observed in this study, where the onset of rigor mortis was faster in fish subjected to greater stress (due to higher density and slaughter due to asphyxiation). However, the fish subjected to thermonarcosis, regardless of the stocking density applied, showed a time to enter rigor mortis similar to that of the control treatment, and this seems to be related to the rapid decrease in physical activity due to thermal shock. For the industry, the extension of the pre-rigor mortis period is important, as this phase is ideal for processing, since filleting the fish in the state of full rigor leads to a reduction in the fillet yield [18]. The generation of H_3_O^+^ ions associated with the production of lactic acid, as well as the collapse of ATP reserves, result in a reduction in muscle pH [63] in several species of fish, such as *Salmo salar* L. [64], *Anguilla Anguilla* [65], and *Pagrus pagrus* [66]. In the present study, the experimental management did not affect the pH after 24 hours of Nile tilapia fillets. In fact, several works indicate that the final pH (after 24 hours) in tilapia fillets is not affected by different levels of pre-slaughter stress, although this stress affects other meat quality characteristics [10, 12, 24].

In this study, instrumental fillet coloration was affected by pre-slaughter stress. Color is generally considered the most important sensory characteristic when evaluating the appearance of a food, especially meat, it is the main criterion by which consumers evaluate the quality and acceptability of meat [67]. The color dependent on the chemical state of the pigment myoglobin, which is responsible for the reddish-purple color, changes due to the presence of natural pigments. These pigments are unstable and participate in different reactions. As a result, the change in color of a food is an indicator of chemical and biochemical changes that may occur during processing and stocking [68], in addition to variables such as pre-slaughter conditions, state of oxygenation and oxidation of the muscle [69]. In this study, fish with the highest density presented meat with a lower intensity of red, a factor that can be attributed to the absence of petechiae or hemorrhages. The lack of pigmentation in fish muscles is desired by consumers [68].

The animals subjected to asphyxiation had fillets with higher brightness and lower intensity of yellow. These color changes are caused by the reduction of soluble muscle proteins in the flesh of stressed fish, these proteins suffer denaturation becoming insoluble and causing a loss of water from the meat, which result in changes in the reflection of light from the surface, thus causing changes in brightness (L*), in the intensity of red (chroma a*) and in yellow (chroma b*) [20]. For cod (*Gadus morhua*), the yellow color in non-stressed fish fillets was significantly higher than in stressed fillets [70].

High pre-slaughter stress in *O. niloticus* subjected to high stocking densities and slaughtered by asphyxiation resulted in less firm fillets compared to fish kept at lower densities and stunned by thermonarcosis. This study highlighted that different slaughter methods significantly influenced stress response, flesh quality, and post-mortem oxidation in *Larimichthys crocea*. Excessive softening of fish meat generally makes it too fragile for processing [71] and affects consumer acceptance [7]. Pre-slaughter stress in several fish species, including *Gadus morhua* [70, 72], *S*. *salar L*. [73], and *O. niloticus* [7], results in very tender fillets. Mechanisms linked to muscle softening due to acute pre-slaughter stress include a decrease in initial meat pH [73], increased protein denaturation, oxidative stress, protein oxidation [7], and subsequent biological changes such as protein fragmentation or aggregation and decreased solubility, all of which affect meat quality [74].

Water holding capacity (WHC) is another quality parameter affected by pre-slaughter stress. In the present study, slaughter by asphyxiation led to the development of fillets with lower WRC in relation to stunning by thermonarcosis, as well as by slaughter by section of the pith (control). Fluid retention is economically important, as these losses that cause weight loss and exudates are not attractive [75]. Previous study with *O. niloticus* reported a decrease in WHC according to the level of stress [24], and this can be explained by changes in protein properties that are important for both WHC and textural properties in the muscle [70]. The ability of muscle to retain water is affected by several factors, such as pH, postmortem protein oxidation, proteolytic activity of meat tenderizing enzymes, and cross-linking of myofibrillar proteins [76]. Excessive loss of water is not desirable for either the industry or the consumer, as it causes losses in the sensory characteristics of the meat, such as texture, tenderness, color and juiciness, making it unattractive, in addition to reducing its yield and nutritional value [77].

Cooking Weight Loss (CWL) of fillets was also affected by pre-slaughter stress, with fish subjected to 300 kg m^−3^ and asphyxiation stunning showing the highest water losses after cooking. Slaughter by bleeding the silver carp gills resulted in a higher CPP compared to slaughter by cranial percussion and stunning by thermonarcosis [78], demonstrating that high stress during pre-slaughter and slaughter can also affect losses due to the baking.

The alterations observed in the instrumental quality of the fillets also resulted in changes in one of the sensory attributes, in which the fillets that underwent asphyxiation presented inferior results in relation to the stunning due to thermonarcosis, the attributes of juiciness. In a study done with cod, it was found that less stressed fish also obtained a higher score in this attribute [79]. The lowest average obtained for the juiciness attribute, in fish submitted to asphyxia, may be related to the lower WHC of these fillets, since this parameter brings the sensation of juiciness during chewing [80]. In addition, although there was no statistical difference, there appears to be a pattern in which the firmness of the fillets is lower in the asphyxiation method than in the other stunning method, showing less firmness, and it is known that very soft fillets are highly undesirable and can have a major impact on consumer acceptability [7, 81].

Although the density of 50 kg m^−3^ leads to an increase in slaughter costs in the aquaculture industry, further studies must be carried out in order to elucidate whether this generated product can provide an increase in shelf life that can reduce the impacts economically.

## Conclusions

It is concluded that the stocking density of 50 kg m^−3^ during pre-slaughter, combined with the thermonarcosis stunning method, induces a lower incidence of muscle and heart damage, which is reflected in a longer permanence in pre rigor mortis that results in filets with enhanced sensory profiles.
